# Intraoral Scanners in Orthodontics: A Critical Review

**DOI:** 10.3390/ijerph19031407

**Published:** 2022-01-27

**Authors:** Isidora Christopoulou, Eleftherios G. Kaklamanos, Miltiadis A. Makrygiannakis, Ilias Bitsanis, Paula Perlea, Apostolos I. Tsolakis

**Affiliations:** 1 Department of Orthodontics, School of Dentistry, National and Kapodistrian University of Athens, 11527 Athens, Greece; mimak90@hotmail.com (M.A.M.); ilbitsani@gmail.com (I.B.); apostso@otenet.gr (A.I.T.); 2 Department of Orthodontics, Hamdan Bin Mohammed College of Dental Medicine (HBMCDM), Mohammed Bin Rashid University of Medicine and Health Sciences (MBRU), Dubai 505055, United Arab Emirates; kaklamanos@yahoo.com; 3 Department of Dentistry, School of Medicine, European University Cyprus, Nicosia 2404, Cyprus; 4 Department of Endodontics, Faculty of Dental Medicine, “Carol Davila” University of Medicine and Pharmacy Bucharest, 020021 Bucharest, Romania; decanatmedicinadentara@gmail.com; 5 Department of Orthodontics, Case Western Reserve University, Cleveland, OH 44106, USA

**Keywords:** digital impression, intraoral scanning, intraoral digital impression, intraoral scanner, intraoral digital scanner

## Abstract

Background: The use of digital technology has exponentially increased over recent years. Intraoral scanners, especially, have gained traction within orthodontics. The objective of the present review is to investigate the available evidence to create an up-to-date presentation of various clinical aspects of intraoral scanners in orthodontics. Methods: Search without restrictions in seven databases (Pubmed, CENTRAL, Cochrane Reviews, Scopus, Web of Science, Clinical Trials, Proquest) since inception, and hand searching until October 2020, were conducted. Results: The majority of studies were either cross-over or parallel group studies. The accuracy and reproducibility of intraoral scanners, in comparison to conventional methods, were investigated in several studies, with controversial results. The duration of the procedure did not report any clear outcome in favor of any method. Patients seem to prefer intraoral scanning, even though numerous studies point out the importance of operators’ experience and skills. Conclusions: Despite the innovations that intraoral scanners have brought in orthodontic clinical practice, there are still some challenges and limitations in their use. The majority of existing limitations may be overcome with experience and good clinical skills. More high-quality studies need to be conducted so that clinicians can have a clear image of this new technology.

## 1. Introduction

Digital technology started to make its way into dental and orthodontic offices with the introduction of Computer-Aided Design/Computer-Aided Manufacturing (CAD/CAM) in 1973 [1,2]. New inventions, such as intraoral scanning, cone beam computed tomography (CBCT), and 3D printing, have introduced the digital era in dentistry [1]. Intraoral scanners constitute a significant chapter in this evolution, with a very promising future ahead [3]. They are devices manufactured to capture direct optical impressions in dentistry [2,3,4,5]. A prototype device for digital impression was presented for restorative dentistry in 1987 by Sirona Dental Systems (Chairside Economical Restoration for Esthetics Ceramics (CEREC) system), and the first in office digital impression system capable of full arch scanning (Cadent iTero) was made available in the dental market in 2008 [6,7]. Since then, technology has greatly evolved, and several companies have launched different models of intraoral scanners [5].

Digital impressions have brought innovation to impression taking, and have partially sidelined the conventional methods (alginate and PVS (Polyvinyl Siloxane)) [8]. Intraoral scanners can offer significant advantages, such as reduced patient discomfort, time efficiency, simplification of clinical procedures, and the benefit of capturing and storing highly accurate information [9]. Their use in the domain of orthodontics is growing wider in recent years, thanks to their potential to perform full arch scanning, indirect bonding, and digitally fabricate fixed orthodontic appliances [1]. They also facilitate orthodontic diagnosis and treatment planning, offering easy and fast electronic transfer of data, immediate access, and reduced storage space requirements [10]. Intraoral scanners provide the orthodontist with numerous applications, such as measurements of arch width and length, tooth size, transverse dimensions, Bolton discrepancy, overjet, and overbite, which are obtained with a remarkable accuracy and efficiency [1]. The user can also create a digital diagnostic set-up, and simulate a proposed treatment plan [11], giving the opportunity to establish a more substantial relationship between patient and orthodontist [2]. Digital dentistry and intraoral scanners, specifically, are also transforming the relationship between the dentist and the dental laboratory [12]. Treatment ergonomics are ameliorated, since digital impressions offer a digital information flow, which will transit within the office and outwards towards patients and dental laboratories [13,14]. The potential to easily transfer digital data to the dental technician has a triple benefit: avoiding shipping time, reducing costs, and facilitating the visualization process, resulting in fewer inaccuracies [9].

However, there are several drawbacks that currently limit the use of intraoral scanners in clinical practice. The high cost of the intraoral scanner, the annual fees, along with the associated hardware and software, and the need to be connected to a network, do not allow the existence of an intraoral scanner in every orthodontic office [9,15,16]. In addition, there is a learning curve for adopting IOS in the dental clinic, and this aspect must be considered [9,17]. Clinicians with a greater affinity for the world of digital technology will probably find it very easy to adopt IOS in their practice. Conversely, older clinicians with less experience could find using these devices and the related software more complex [18]. Moreover, the IOS, currently available commercially, differ in terms of accuracy and time efficiency; therefore, the contemporary devices may have wider indications for clinical use, whereas the oldest have fewer clinical indications [9,19,20,21]. This is an important aspect to be considered before purchasing an IOS, in addition to other important features [19,21].

Over the past years, there has been a dramatic increase in published studies concerning intraoral scanners and their uses. Two recently published systematic reviews on intraoral scanners focus on the accuracy [22], and the patient-reported experiences and preferences with intraoral scanners [23]. However, until today, except from the systematic reviews examining only specific aspects of intraoral scanners, the reviews that investigate several parameters simultaneously are scarce and inconclusive. For these reasons, we decided to perform a critical review, using a standard framework, and attempted to answer, with a critical eye, a series of questions concerning intraoral scanners that may be of interest to the reader. The objective of the present review is to investigate the available evidence to create an up-to-date presentation of various clinical aspects of intraoral scanners in orthodontics.

## 2. Materials and Methods

Regarding the eligibility criteria, we placed no restrictions on study design, year of publication, or study location. The included papers should be studies evaluating the clinical parameters of intraoral scanners, such as accuracy, reproducibility, duration, and operators’ experience, as well as person-reported information, such as perception of time, comfort, and preferences about intraoral scanners. Animal studies; non-comparative studies; and studies involving dry skulls, phantoms, and reference models were excluded.

**Figure 1:**
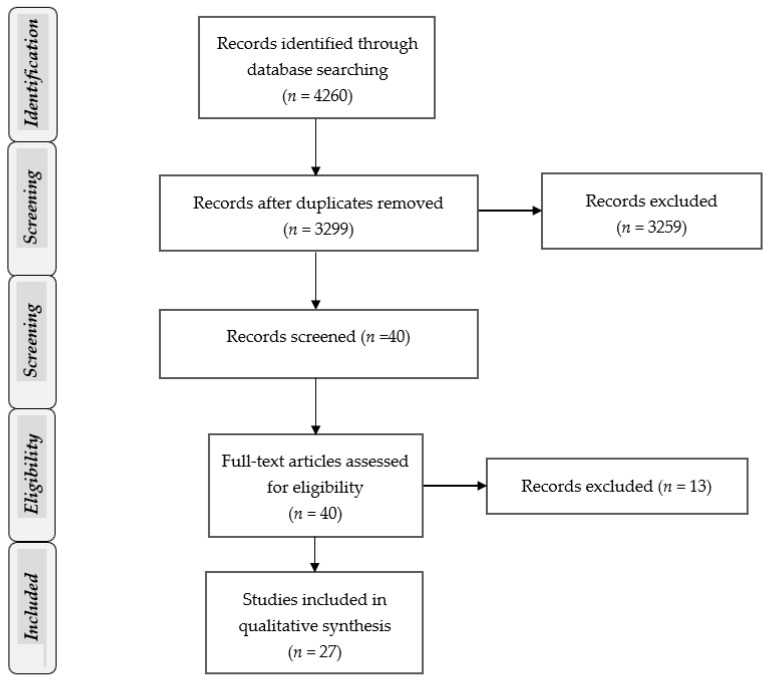
Flowchart of records.

Overall, seven electronic databases (Pubmed, CENTRAL, Cochrane Reviews, Scopus, Web of Science, Clinical Trials, Proquest) were searched from inception until October 2020 by using key items. The detailed search strategies for each database are presented in Supplementary Table S1. The flowchart of records through the reviewing process is shown in Figure 1. We also conducted hand searching of gray literature material. No restriction was placed on date and publication status. In addition, efforts to obtain additional or ongoing trials were made, and the reference lists of all eligible studies, as well as relevant reviews, were searched.

## 3. Results

### 3.1. Accuracy

As far as the accuracy of intraoral scanners is concerned, the reported results proved to be contradictory [24,25,26,27,28,29,30,31,32,33,34,35]. Specifically, when linear measurements are examined, digital models from a Lythos scanner can be as accurate as digital models from conventional impressions [26]. This means that the dimensions of the teeth on the occlusal aspect can be very precisely represented in a digital image, enabling clinicians to work on treatment planning for patients, and to decide on space requirements [26]. In addition, some clinical trials demonstrate that the in vivo precision of guided scanning procedures is superior to conventional impression techniques (alginate) [27]. Duvert et al. [28] disagreed, reporting that under ideal circumstances, the precision of impressions obtained by intraoral scanners (i-Tero, Lythos, TRIOS) is of lower quality than that obtained by conventional materials and digitized by extraoral scanners.

Grunheid et al. [24] and Sfondrini et al. [25] confirmed the potential of intraoral scanning to acquire data as accurate as alginate impressions for orthodontic applications, whereas other studies concluded that conventional impression materials provide significantly higher precision than digital impression systems [29,30]. However, it should be highlighted that patient-related factors, such as the intraoral conditions, may influence the scanning process [31]. In addition, the scanning of the maxilla seems to be less accurate than the scanning of the mandible [31]. Rhee et al. [32] tried to be more specific, stating that the two-dimensional deviations in conventional impressions were smaller than those recorded with intraoral scanners. When comparing the 3D and 2D dimensions, the second premolar demonstrated the greatest deviations, showing significantly greater three-dimensional (3D) deviations than those of the second molar [32].

When comparing 3D models obtained by CBCT to 3D models obtained by intraoral scanning, it was shown that models made with intraoral scanning methods provided statistically and clinically acceptable accuracy for all direct and indirect dental measurements, whereas CBCT models presented a tendency to underestimate dental measurements in the lower arch, although still within the limits of clinical acceptability [33].

Based on our clinical experience, in cases with crowding, moderate or severe, it is not yet clarified whether the digital method is more accurate than the conventional, and further research is needed to decide the credibility of digital measurements. The recent technological advancements in IOS aim to improve the accuracy and reproducibility of the recorded measurements, facilitating both diagnosis and treatment planning.

When comparing two intraoral scanners to each other, Kierl’s study suggests that the accuracy of both the iTero Element and the 3Shape TRIOS intraoral scanners was considered to be clinically adequate [34]. In another study, it was observed that TRIOS had a higher accuracy than CEREC [35].

### 3.2. Reproducibility

The reproducibility and reliability of intraoral scanners were examined in several studies [28,36,37,38,39,40], with interesting results, and they proved to be excellent [28,36,37]. When comparing in vivo and ex vivo scanning, Duvert et al. [28] and Sun et al. [36] supported the idea that the reproducibility of in vivo scanning was comparable with the reproducibility of ex vivo scanning, even though it presented a slight difference. In regards to linear measurements, the same level of reproducibility was reported for both conventional and digital models [38].

With the purpose of examining reproducibility, validity, and reliability, all together, Wiranto et al. [39] demonstrated that both intraoral scanning and CBCT scanning of alginate impressions are valid and reproducible. In a more recent study, Kirschneck et al. [40] suggested, as well, that even though indirect alginate impressions proved to show a statistically significant higher reproducibility than direct digital impressions, all procedures proved to be clinically equivalent for diagnostic purposes when comparing models obtained by a Lythos intraoral scanner, alginate, and polyether.

### 3.3. Duration

The time needed for a digital impression obtained by an intraoral scanner in comparison to the time needed for a conventional impression is a parameter investigated in numerous studies [2,24,25,41,42,43], with contradictory results. Burhardt et al. [41] reported no significant differences in chairside time among specific groups (1st group alginate, LAVA, CEREC; 2nd group LAVA, CEREC, alginate; 3rd group CEREC, alginate, LAVA-impressions; all made in that order). Chairside time proved to be less with alginate, followed by CEREC and LAVA [41]. LAVA had the longest chairside time [41]. Other studies have also confirmed that the chairside time needed for alginate impressions is shorter compared to the time needed for the digital ones [2,24,42]. Moreover, it has been claimed that when comparing intraoral scanning, alginate, and PVS impressions, alginate impressions still require the least time [43]. Sfondrini et al. [25] interestingly stated that new generation powder-free scanners reduce both chairside and processing times.

Based on our experience, digital impressions are time-efficient, as they enable the reduction of working times when compared to conventional impressions. The possibility to capture a full-arch scan in approximately less than 3 min, in combination with the time saved afterwards (no need to pour stone casts and fabricate physical plaster models), makes intraoral scanning a time-effective tool in the hands of the orthodontist. However, it should be highlighted that possible intraoral anatomical variations and a limited mouth opening may complicate the procedure, and increase the time needed.

### 3.4. Perception of Time: Patients’ Point of View

Patients’ perception of time for the impression method (conventional or digital) has been examined thoroughly, and it seems that intraoral scanning is more comfortable for patients in terms of time [2,25,41,42,44]. Burhardt et al. [41] suggested that patients preferred the intraoral scanning, although it was perceived to consume more time. Burzynski et al. [42] reported that patients perceived the digital method as faster than expected, and Sfondrini et al. [25] observed that although several patients still considered the time of intraoral scanning too long, they favored the digital method in terms of time perception. Concerning the prescription times, the mean time needed for entering the laboratory prescription for the intraoral impression method proved to be statistically significantly different from the mean application time needed for the conventional impression method [44]. The difference between the mean bite registration time for the conventional technique and the mean bite scan time for the digital technique was also statistically significant [44].

It is true that some negative reactions concerning perception of scanning times have been documented by both patients and clinicians. In our daily clinical practice, we often observe that the patient’s time perception of the procedure, digital or conventional, depends largely on the level of comfort and cooperation between clinician and patient. Patients, most of the time, feel excited by being able to follow the scanning process and seeing their dental arches digitally, ameliorating their perception of time, and making the procedure more pleasant.

### 3.5. Comfort-Feeling of Comfort

In general, digital impression methods prove to be more comfortable in comparison to conventional methods [2,24,25,41,44,45]. In the majority of studies, patients reported more comfortable feelings with digital techniques [2,41,44,45]. When examining comfort following conventional impressions (PVS or alginate) and intraoral scanning, patients stated that the impressions with digital methods and alginate were, statistically, more comfortable than those taken with PVS [43]. The intraoral scanner had a better score in terms of comfort, gag reflex, and breathing difficulty [2]. The use of powder for intraoral scanning is a significant factor in assessing the feeling of comfort, with 64% of patients scanned with LAVA and 74% with CEREC having noticed the powder, and 14% and 18% out of them, respectively, having experienced discomfort related to the powder [41].

The occurrence of gag reflex, as another factor strongly related to the feeling of comfort, is investigated in three studies, all concluding that it is highly reduced—or even absent—when using digital methods [2,41,44]. With the absence of gag reflex, smell/voice and taste/heat were shown to be better and more pleasant with digital impressions [2,43,44].

Indeed, intraoral scanning could be the method of choice in patients with severe gag reflex. However, when assessing the level of comfort, the current studies did not take into consideration the consistency and the quantity of the conventional impression material, and, along with that, the possibility of having to retake the conventional or digital impression in case of mistakes or inaccuracies.

### 3.6. Experience of the Operator

The experience of the operator proved to be a significant factor in the general assessment of intraoral scanners [46,47,48]. In the study of Kim et al. [47], it is mentioned that the learning rate for iTero was faster, and the average scanning time for iTero was greater, than that for TRIOS. The learning rate for TRIOS was slow, but the mean scanning time was less, and was not influenced by clinical experience [47]. It is also interesting that the learning rate for TRIOS differed according to the participant’s intraoral characteristics [47]. Lim et al. [48] suggested that practice, clinical experience, and the scanned area all affected the fidelity of scanned images. Consequently, users require practice opportunities for effective clinical application [48]. As far as hygienists are concerned, their views changed positively after training in the use of intraoral scanners [46]. Mangano et al. [9] stated, in their review article, that there is a learning curve for adopting intraoral scanning in the dental clinic, and this aspect must be considered.

Unfortunately, objectivity cannot be easily achieved, as it is shown that clinicians with less experience in the digital field and more familiarity with conventional methods may find the use of the devices and the related software more complex, whereas dental students, being more familiar with digital methods, seem to attain clinical competence faster with intraoral scanning [49]. In our daily clinical practice, we have observed that the learning curve is different for each clinician depending on age, previous or no experience with digital devices, the ability to easily adapt to new technologies, and also the level of cooperation with patients.

### 3.7. Preference

#### 3.7.1. Preference of Method: Conventional or Digital?

In general, the majority of patients preferred the digital methods [2,44,45,50]. Mangano et al.’s and Yuzbasioglu et al.’s questionnaires examined the same parameters, and both studies concluded that all patients (100%) chose the intraoral scanners [2,44]. Conversely, the study of Burzynski et al. [42] suggests that participants who experienced conventional alginate methods showed less preference towards digital methods, whereas i-Tero and TRIOS participants stated that they would rather choose an orthodontist with digital scanners. In contrast, Grunheid et al. [24], suggested that participants preferred the alginate methods over the intraoral scanners.

It is stated that, when used for Invisalign treatments, intraoral scanning is a more attractive option for patients [51]. Moreover, examining the orthodontists’ point of view, in a questionnaire study, it is reported that when orthodontists were asked if they planned to switch to digital study models in the future, 34% said yes, 29% said no, and 37% were undecided [52]. However, the high cost of the hardware and software, as well as the lack of training, currently limit their use in the field of orthodontics.

#### 3.7.2. Preference of Scanner

Operators generally preferred the TRIOS intraoral scanner over iTero concerning the difficulty of use and the clinical usefulness [46]. Conversely, Park et al. [52] found in a questionnaire study among practicing orthodontists that, among digital users, 56% reported using intraoral scanners, with iTero being the most popular (86%). It is also mentioned that, as per patients’ comfort and convenience, iTero was rated higher in comparison with TRIOS [46]. When comparing LAVA and CEREC, 44% of patients preferred CEREC, and 39% did not differentiate between the two scanners [41].

Table 1 and Table 2 summarize the clinical aspects investigated in the studies included in the present review. Supplementary Table S2 summarize the characteristics of the clinical studies included in the present review.

## 4. Discussion

The purpose of a dental impression is to copy a patient’s intraoral situation, transforming it into a model. Obtaining a model of good quality, true to the original, is extremely important for the success of a treatment. Different types of materials and impression techniques have been used over the years to achieve the desired accuracy [53]. Concerning the limitations of existing studies, it is true that only few have evaluated complete-arch scans acquired directly in the patient’s mouth, and further studies should examine whether accuracy of digital impressions for whole upper jaws is clinically acceptable [54]. It should also be pointed out that accuracy and reliability were comprehensively evaluated in only one paper [37]. Additionally, there is a difference in the statistical analysis between the studies, as Naidu et al. [37] and Wiranto et al. [39] use the t-test, whereas Grunheid et al. [24] use the Bland–Altman plot.

As far as reproducibility is concerned, crowding seems to play an important role in the results because of the accumulation of errors that occurred in single measurements [38]. The differences found for crowding seemed not to be dependent on the severity of crowding, concluding that digital models can also be used for cases with crowding of over >4.5 mm [38]. However, further studies are warranted, and clinicians should keep in mind that crowding measured by digital models tends to be lesser than that measured by cast models, and this should be considered during clinical application [38].

In regards to the efficiency and efficacy of intraoral scanning, which are associated with the above-mentioned parameters, Naidu et al. [37] claimed that although its efficacy has already been tested, its efficiency has not. In the study of Burzynski et al. [42], it is reported that intraoral scanners have comparable efficiency with conventional impression methods depending on the type of the scanner. However, other authors supported the view that the digital impression technique was more efficient than the conventional impression method, regardless of the scanner type [44]. Due to the small number of studies, it seems reasonable to conclude that further high-quality studies, with an adequate sample of patients and different intraoral scanners, are required to examine the efficacy and efficiency.

In general, in orthodontics and in all fields of dentistry, a short duration of clinical procedures is the key to a successful and comfortable procedure for both patient and dentist. The scanning times measured in the published studies varied largely. It should be noted that, in order to be objective, it is important to compare the same malocclusions in patients with the same level of tolerance, as it is possible that different intraoral situations (e.g., rotations) and different behavior patterns may influence the duration of the procedure. Moreover, the level of patient’s tolerance is frequently associated with the age of the participant. With respect to the emerging maturity of children and adolescents, they seem to be less tolerant and objective in this kind of assessment. Consequently, it is essential to conduct studies with participants of different age groups, and with adults always included.

Comfort seems to be more easily achieved with intraoral scanners. In the majority of studies, the feeling of comfort is analyzed with the use of VAS (Visual Analog Scales). VAS are a useful tool for assessing patients’ point of view, especially with computerized versions of VAS-A, which present additional advantages, including facilitated and accurate data collection and analysis [55]. However, it is important to mention that the parental presence, the age of the patients, and, along with that, the level of maturity, are still considered as potential confounding factors.

Concerning the experience of the operator, it should be kept in mind that it is still unclear whether one scanning strategy is better than another, as manufacturers provide little information about their scanning strategies [9]. This is an aspect that needs to be researched in-depth in the future, as it is possible that different devices with different scanning strategies would produce different measurements and results [9].

Furthermore, even though experience is gained, and orthodontists seem to be more interested in intraoral scanners, their high cost is a parameter that cannot be neglected. Different manufacturing companies have different policies, and it is important for the clinician to be fully informed of the associated costs and the annual management fees before purchasing an intraoral scanner [4,5,9,22,56]. It is essential that the new intraoral devices should be cushioned by integrating into the clinical workflow across the dental disciplines [9].

Regarding emerging technologies, such as IOS, it is always valuable to compare the findings of the older and newer publications within a period of time. Systematic reviews and reviews offer a better insight into the world of digital impression by reporting the evolution in this field. In general, all the papers included in the present review were published in the last ten years. These studies give clinicians the opportunity to compare the existing devices, and acknowledge the possibilities of the recently launched intraoral scanners, and the improvements that have been achieved or are yet to be accomplished. However, it is true that as technology constantly progresses and new devices become commercially available, it is better to focus on recent publications when thinking of purchasing an intraoral scanner. The intraoral scanners that were mentioned and examined in the clinical studies included in the present review are: the TRIOS Classic (3Shape, Copenhagen, Denmark), the TRIOS 3 Mono Intraoral Scanner (3Shape, Copenhagen, Denmark), the LAVA C.O.S. (3M ESPE, St. Paul, MN, USA), the CEREC Omnicam (Sirona Dental Systems, Bensheim, Germany), the iOC intraoral scanner (Cadent, Carlstadt, NJ, USA), the CS3600 Carestream (Dental, Rochester, NY, USA), the iTero Element intraoral scan (Align Technologies, San Jose, CA, USA), and the Lythos (Ormco, Orange, CA, USA).

Based on our experience, intraoral scanning could be the ideal method of impression taking, and the future of impressions in orthodontics. These devices offer numerous applications with a remarkable accuracy, which is constantly ameliorating from the manufacturers. They also facilitate the clinical procedure, in terms of time and comfort for both patients and dentists. Digital impressions can be used as a starting point for the realization of a whole series of customized orthodontic devices, among which, aligners seem to be the mostly used. As aligner technology constantly progresses, intraoral scanners become more and more necessary in an up-to-date orthodontic office. In the coming years, it will be possible that almost all orthodontic appliances will be designed digitally with impressions from an intraoral scanner, so they will be entirely personalized and adapted to the patient’s specific intraoral needs. The 3D evolution, and especially intraoral scanners, will totally change the ‘way of thinking’ in the field of orthodontics, modernizing the role of the ‘traditional’ orthodontist in diagnosis and treatment processes. The ideal would be a combination of conventional and digital impression methods in daily orthodontic clinical practice in an attempt to learn from each method’s advantages and disadvantages.

## 5. Conclusions

In conclusion, according to the available data, it is important to embrace the digital era in orthodontics, but always with due care. Even though intraoral scanning is at its very beginning, it has shown a great potential, offering the orthodontist various possibilities, and modernizing the clinical practice. Nevertheless, despite the innovations that intraoral scanners have brought in orthodontic daily practice, there are still some challenges and limitations in their use. The majority of existing limitations may be overcome with experience and good clinical skills. However, more high-quality studies are essential for orthodontists to be able to correctly interpret the data, appraise the new findings, compare them to the existing evidence, and form an up-to-date, objective opinion.

## Figures and Tables

**Table 1:** Clinical aspects of conventional methods and intraoral scanning.

	Conventional Methods	Intraoral Scanning	Conflicting Results
Accuracy			√
Reproducibility	√	√	
Validity	√	√	
Efficiency			√
Time			√
Perception of time			√
Feeling of comfort		√	
Person-reported preferences and experiences			√

√: Greater acceptability of the specific method of impression. When this symbol is in two boxes, then this specific feature is acceptable in both of them.

**Table 2:** Specific aspects of person-reported preferences and experiences.

	Conventional Methods	Intraoral Scanning	Conflicting Results
Stress		√	
Fear		√	
Feeling of comfort		√	
Pleasant feelings		√	
Queasiness		√	
Gag reflex		√	
Taste/smell/heat		√	
Painless		√	
Breathing difficulty		√	
Dry mouth		√	
Powder and related feelings			√
Perception of time			√
Tooth/gingival sensitivity		√	
Overall opinion/preference			√

√: Greater acceptability of the specific method of impression.

## Supplementary Materials

The following supporting information can be downloaded at: https://www.mdpi.com/article/10.3390/ijerph19031407/s1, Table S1. Search strategy for each database and relative results; Table S2. Characteristics of the clinical studies included in the review. References [2,24,25,26,27,28,29,30,31,32,33,34,35,36,37,38,39,40,41,42,43,44,45,46,47,48,50] are cited in the Supplementary Materials.

## Abbreviations

PVS	Polyvinyl Siloxane
IOS	Intraoral Scanning
VAS	Visual Analog Scales
