# Supplementary material for: Intraoral Scanners in Orthodontics: A Critical Review
Source: Int J Environ Res Public Health. 2022 Jan 27;19(3):1407. doi: 10.3390/ijerph19031407 (PMC8834929; doi:10.3390/ijerph19031407)
Supplement: Supplementary file 1 [file ijerph-19-01407-s001.zip › ijerph-1541452-supplementary.pdf]

Table S1. Search strategy for each database and relative results.

Database	Search Strategy	Results
**Pubmed**	(digital impression) OR (intraoral digital impression) OR (intraoral scanning) OR (intraoral scanner) OR (intraoral digital scanner)	**2877**
**CENTRAL**	digital impression) OR (intraoral digital impression) OR (intraoral scanning) OR (intraoral scanner) OR (intraoral digital scanner) in Record Title—(Word variations have been searched)	**39**
**Cochrane Reviews**	digital impression) OR (intraoral digital impression) OR (intraoral scanning) OR (intraoral scanner) OR (intraoral digital scanner) in Record Title—(Word variations have been searched)	**0**
**Scopus**	TITLE (digital impression) OR (intraoral digital impression) OR (intraoral scanning) OR (intraoral scanner) OR (intraoral digital scanner))	**619**
**Web of Science**	TITLE: (((digital impression) OR (intraoral digital impression) OR (intraoral scanning) OR (intraoral scanner) OR (intraoral digital scanner))) Timespan: All years. Databases: WOS, KJD, RSCI, SCIELO, ZOOREC. Search language = Auto	**565**
**Clinical Trials**	(digital impression) OR (intraoral digital impression) OR (intraoral scanning) OR (intraoral scanner) OR (intraoral digital scanner)	**131**
**Proquest**	Ti((digital impression) OR (intraoral digital impression) OR (intraoral scanning) OR (intraoral scanner) OR (intraoral digital scanner)) in Full text	**29**

Table S2. Characteristics of the clinical studies included in the review. [2,24–48,50]

Studies	Participants	Interventions-Comparison	Outcomes assessed
**Burhardt et al. [41] (2016)**	Thirty-eight orthodontic patients (22 F, 16 M; 10–17 y, median 12 y)<br>Criteria (+) good physical/mental health; (−) clefts, craniofacial anomalies, dental fear, gag reflex	Group 1: ALG; IOS (LAVA); IOS (CEREC)<br>Group 2: IOS (LAVA); IOS (CEREC); ALG<br>Group 3: IOS (CEREC); ALG; IOS (LAVA)	• **Comfort:** (ALG < CEREC) for mx<br>• **Duration:** (ALG > CEREC > LAVA)<br>• **Time perception:** (ALG < CEREC)<br>• **Preference of method:** (IOS > ALG)
**Burzynski et al. [42] (2018)**	One hundred-eighty orthodontic patients (104 F, 76 M; 8–56 y, median 15 y)<br>Criteria (+) healthy, english-speaking, seeking ortho Tx or under ortho Tx; (−) mental disabilities, clefts, craniofacial anomalies, syndromes	Group 1: IOS (iTero)<br>Group 2: IOS (TRIOS)<br>Group 3: ALG	• **Duration:** (ALG > IOS)<br>• **Time perception:** (ALG > iTero)<br>• **Comfort:** (iTero > TRIOS/ALG)<br>• **Importance of IOS:** (iTero > TRIOS / ALG)<br>• **Preference of method:**<br>(IOS participants: IOS > ALG)<br>(ALG participants: ALG > IOS)
**Christou [26] (2015)**	Thirty orthodontic patients (> 19 y)<br>Criteria (+) medically healthy, good oral hygiene, permanent dentition; (-) dentofacial deformities, permanent retainers bonded, extensive restorations, scans not completed, PVS impr not in excellent condition	Each patient: PVS; IOS (Lythos)<br>Extra oral digitization of PVS impr using extra-oral scanner GOM ATOS	• **Accuracy:** [IOS (Lythos) = PVS - linear measurements]
**Darroudi et al. [43] (2017)**	Ten patients (> 18 y)<br>Criteria (+) full permanent dentition, good general/mental health/oral hygiene; (−) periodontal abnormalities, prostheses, bad quality restorations, orthodontic appliances/fixed retention, poor experience with impressions	Each patient: ALG; PVS; IOS (TRIOS)	• **Preference of method:** (IOS > PVS)<br>• **Time perception:** (IOS > PVS)<br>• **Duration:** (ALG > IOS, PVS)
**Duvert et al. [28] (2017)**	One patient<br>Criteria (+) complete natural denture	IOS (Lythos, iTero, TRIOS); PVS<br>Extraoral digitization of PVS impr	• **Accuracy:** (PVS > IOS)<br>• **Reproducibility:** (PVS > IOS)
**Ender et al. [29] (2016)**	Five patients<br>Criteria (+) complete dentition	Each patient:<br>Conv: POE; VSE; VSES; ALG<br>Digit: CEREC Bluecam; CEREC Omnicam; iTero; LAVA; True	• **Accuracy:** [Conv (POE, VSE, VSES) > IOS]<br>(IOS > ALG)

		Definition; 3Shape TRIOS; 3Shape TRIOS color	
**Flugge et al. [31] (2013)**	One patient	IOS (iTero); POE<br>Extraoral digitization of conv models using D250	• **Accuracy:** (POE > IOS), (D250 > iTero)
**Glisic et al. [45] (2019)**	Fifty-nine patients (28 F, 31 M; 9–15 y)<br>Criteria (+) indication for ortho Tx; (−) previous orthodontic treatment, craniofacial syndromes	Each patient: ALG; IOS (TRIOS)	• **Comfort:** (TRIOS > ALG)<br>• **Preference of method:** (IOS > ALG)
**Grünheid et al. [24] (2014)**	Fifteen patients (9 F, 6 M; 19.5 +_ 9.9 y)<br>Criteria (+) full permanent dentition from FM to FM; (−) supernumerary teeth, mental, emotional, developmental disabilities, cleft lip/palate, craniofacial anomalies, epilepsy/seizures, anticonvulsants	Each patient: ALG; IOS (LAVA)	• **Accuracy:** (ALG = IOS)<br>• **Preference of method:** (ALG > IOS)<br>• **Duration:** (ALG > IOS)
**Kierl [34] (2018)**	Twenty-five patients<br>Criteria (+) student volunteers from the OU College of Dentistry, all permanent teeth, FM to FM in both dental arches, minimal dental crowding (< 4 mm)	Each patient: IOS (iTero, TRIOS); ALG	• **Accuracy:** (IOS > ALG); (TRIOS = iTero)
**Kim et al. [47] (2016)**	Four patients and twenty-nine dental hygienists as operators<br>Criteria (+) no experience using 3D digital IOS, currently working in clinical practice were recruited	Each participant: IOS (iTero, TRIOS)	• **Duration:** (TRIOS > iTero)<br>• **Easier-faster learning rate:** (iTero > TRIOS)
**Kirschneck et al. [40] (2018)**	Twenty patients (18–30 y)<br>Criteria (+) complete permanent dentition, no current caries activity, gingivitis, periodontal disease or other oral pathology; (-) systemic diseases, congenital anomalies and syndromes, current orthodontic treatment, allergies to any component of the applied impression materials.	Each patient: POE; ALG; IOS (Lythos)<br>Extraoral digitization of conv models using both the 3Shape D810 (3shape) scanner and the Atos II Triple Scan (GOM)	• **Reproducibility:** (ALG > IOS) (D810 = Atos II Triple Scan)
**Kuhr et al. [30] (2016)**	Fifty patients (25 F;25 M)<br>Criteria (+) complete lower dental arch (fully dentate or fixed restorations)	Each patient: POE; IOS (CEREC, TRIOS, 3M True Definition)	• **Accuracy:** [POE > IOS (3M True Definition, TRIOS, CEREC)]
**Lim et al. [48] (2017)**	One patient and twenty dental hygienists (20 F) as operators<br>Criteria (+) > 3 y of experience in dental clinical practice (group 1: 3–5 y; group 2: > 6 y)	IOS (iTero, TRIOS) for each of the operators	• **Easier-faster learning rate:** (TRIOS = iTero)**;** affecting the fidelity of scanned images
**Mangano et al. [2] (2018)**	Thirty patients (15 F, 15 M; 7–16 y);<br>Criteria (+) good oral health/hygiene; (−) periodontal disease, history of ortho TX, prostheses	Each patient: ALG; IOS (CS3600)	• **Comfort:** (ALG < IOS)<br>• **Duration:** (ALG > IOS)<br>• **Time perception:** (ALG < IOS)<br>• **Preference of method:** (IOS > ALG)
**Naidu et al. [37] (2013)**	Thirty patients<br>Criteria (+) full complement of permanent teeth, no missing or heavily restored teeth, no teeth with large carious lesions or enamel defects	Each patient: ALG; IOS (iOC)	• **Reproducibility:** (IOS > ALG)
**Nedelcu et al. [35] (2017)**	Five patients<br>Criteria (+) only second premolar missing, residual spacing in the premolar to premolar area.	Each patient: IOS (3M True Definition, CEREC, TRIOS); POE	• **Accuracy:** (IOS = POE); (TRIOS > CEREC)
**Park et al. [46] (2015)**	Twenty-four patients (dental hygienists)	Each participant: IOS (iTero, TRIOS)	• **Preference of scanner:** (TRIOS > iTero) (as operators)

	Criteria (+) no previous experience with IOS	Each participant served both as operator and patient	
**Rhee et al. [32] (2015)**	Twenty-four patients<br>Criteria (+) no periodontitis or temporomandibular joint disease	Each patient: PVS; IOS (TRIOS)<br>Extraoral digitization of conv models using 3D laser scanner	• **Accuracy:** (PVS > IOS) 3d deviations: (IOS, dual arch > full arch, dual arch), 2d deviations (PVS > IOS)
**San Jose et al. [33] (2017)**	One hundred patients (53 F, 47 M; 21.6 to 29.2 y)<br>Criteria (+) permanent dentition from FM to FM, initial records included a CBCT, an intraoral scan and plaster models; (-) tooth agenesis or extractions, large restorations that could change the mesiodistal diameters of the teeth, teeth with anomalous shapes, infraoccluded teeth.	Each patient: CBCT; IOS (iTero); plaster models<br>Extraoral digitization of conv models using intraoral laser scanner (ILS)	• **Accuracy:** (IOS > CBCT)
**Schott et al. [50] (2019)**	Thirty-one patients (dental students) (22 F, 9 M; 21-31 y)<br>96.8% no previous experience with IOS	Each patient: ALG; IOS (3M True Definition)	• **Preference of method:** (IOS > ALG)
**Sfondrini et al. [25] (2018)**	Fourteen patients (10 F, 4 M; mean 20.4 y)<br>Criteria (+) full permanent dentition; (−) supernumerary teeth; mental or developmental disabilities, craniofacial anomalies, epilepsy, dental fear, gag reflex	Each patient: ALG; IOS (TRIOS)	• **Preference of method:** (IOS > ALG)<br>• **Overall impression:**<br>Gag reflex (IOS > ALG)<br>Equipment size (IOS > ALG)<br>• **Comfort:** (IOS > ALG)<br>• **Time perception:** (IOS > ALG)<br>• **Duration:** (IOS > ALG)
**Sun et al. [36] (2018)**	Twenty patients (>18 y old)<br>Criteria (+) full permanent dentition, no metal or gold crown restorations, without severe crowding and dentofacial deformity	Each patient: IOS (TRIOS); ALG<br>Extraoral digitization of conv models using TRIOS	• **Reproducibility:** (in vivo scanning = ex vivo scanning)
**Wiranto et al. [39] (2013)**	Twenty-two patients<br>Criteria (+) complete permanent dentition from FM to FM in both arches, no fixed orthodontic appliances, no severe crowding in the dentition (\6 mm).	Each patient: IOS (LAVA); ALG (CBCT scans of alginate impr)	• **Reproducibility:** (IOS scanning = CBCT scanning)
**Yoon et al. [38] (2018)**	Forty-six patients<br>Criteria (+) absence of dentofacial deformity or medical problems, absence of previous orthodontic history with fixed appliances, eruption of all permanent teeth, no impacted, missing, or supernumerary teeth, availability of an intraoral-scan model and a cast model.	Each patient: IOS (TRIOS); ALG	• **Reproducibility:** (IOS = ALG)
**Yuzbasioglu et al. [44] (2014)**	Twenty-four patients (12F, 12M; 21.87 ± 2.76 y)<br>Criteria (+) good general/mental health and oral hygiene; (−) periodontal disease, prostheses, moderate to excessive dental anxiety, ortho Tx	Each patient: POE; IOS (CEREC)	• **Comfort:** (CEREC > POE)<br>• **Time perception:** (CEREC > POE)<br>• **Preference of method:** (IOS > POE)<br>• **Duration:** (CEREC = POE)
**Zimmermann et al. [27] (2017)**	Five patients<br>Criteria (+) complete natural dentition	Each patient: IOS (CEREC, Lythos); ALG	• **Accuracy:** (IOS > ALG)

POE : Polyether, VSE: vinylsiloxanether, VSES: direct scannable vinylsiloxanether, ALG: Alginate, PVS: poly-vinyl-siloxane, conv: conventional, digit: digital, mx =maxilla, ortho TX = orthodontic treatment, IOS: intraoral scanner-intraoral scanning, F: female, M: male, y: years, impr: impression, FM: first molar, >: more acceptable, <: less acceptable, (+): inclusion criteria, (-): exclusion criteria.
